# Influence of Different Beverages upon the Elimination of Common Salt, Urea and Sugar in Diabetic Urine

**Published:** 1859-03

**Authors:** 


### TRANSLATIONS BY DR. E. L. HOLMES.

### PATHOLOGICAL CHEMISTRY.

### INFLUENCE OF DIFFERENT BEVERAGES UPON THE ELIMINATION OF COMMON SALT, UREA AND SUGAR IN DIABETIC URINE.

From the "Allgemeine Medicinische Central Zeitung."

Dr. Rosenstein has investigated the manner in which various articles of drink influence the elimination of salt, urea and sugar in diabetes, because he believed the most important means of treatment to be found in the diabetic regimen of the patient; he has arrived at the following results:

1st. After the use of coffee, the elimination of salt and sugar was, on the average, increased, that of urea diminished.

2d. By the use of Bavarian beer, the elimination of salt was considerably increased, that of urea somewhat diminished, and that of sugar also increased, though in a less degree than by the use of coffee.

3d. After the use of wine, of which Dr. R. administered three different varieties, chosen in reference to the amount of alcohol they contained (Bordeaux having the largest, Madeira a less, and pure St. Emillon the least per cent. of alcohol), the elimination of salt was scarcely increased, of urea scarcely diminished, and that of sugar increased. Still the latter was smaller, in proportion as the amount of alcohol in the wine was greater.

4th. By the use of tartaric acid, the elimination of salt was but little increased, that of urea decreased, and of sugar increased.

5th. During a moderate febrile condition, the elimination of salt was normal, that of urea (although increased in proportion to the whole amount of urine), was diminished, and that of sugar much diminished.

In reference to temperature, the observations show: 1st, that the animal heat of the (diabetic) patient is less than that of health; 2d, that there is no observable relation between temperature, and frequency of pulse and respiration; and, 3d, that during a general febrile condition, the temperature is higher than that of a healthy person.—*Virchow's Archiv. of Pathological Anatomy, Physiology, and Clinical Medicine*, xiii. 4, 5.

